# Sero-prevalence of bovine brucellosis in the Bojanala Region, North West Province, South Africa 2009–2013

**DOI:** 10.4102/jsava.v91i0.2032

**Published:** 2020-08-12

**Authors:** Cheryl M.E. McCrindle, Solly N. Manoto, Bernice Harris

**Affiliations:** 1 School of Health Systems and Public Health, Faculty of Health Sciences, University of Pretoria, Pretoria, South Africa

**Keywords:** Bovine brucellosis, sero-surveillance, farming systems, zoonosis, food security, one health

## Abstract

Bovine brucellosis affects food safety, food security and human health in rural communities in the North West Province, South Africa. The World Organisation for Animal Health suggests routine sero-surveillance and vaccination of cattle for control and to prevent zoonotic transmission. Although sero-surveillance and subsidised vaccination have been in place for decades, data from Bojanala have not previously been analysed. The aim of this study was to retrospectively analyse historical data on routine sero-surveillance of bovine brucellosis and state subsidised vaccination, in communal, commercial and dairy cattle in the study area. This was a descriptive, cross-sectional retrospective analysis of records from all adult cows bled by the state veterinary services during routine sero-surveillance for bovine brucellosis, in the Bojanala Region, North West Province, between 2009 and 2013. Fewer communal (*N* = 11 815) and dairy (*N* = 6696), than commercial beef (*N* = 28 251) cows, were tested. Overall herd prevalence (33.33%), differed significantly from individual prevalence (3.18%) in all groups. Communal herds had both the highest herd prevalence (38.8%) and the highest individual prevalence (5.2%). Both herd and individual sero-prevalence were lowest in dairy cattle, possibly because registered dairy herds are routinely tested. Over the 5-year study period, only 24 086 (7.15%) of the 342 500 cows eligible for free vaccination, were vaccinated. The annual number of cattle tested was highly variable. Dairy cattle that were regularly tested had a significantly lower herd and individual prevalence. Herd prevalence would be useful for spatial mapping, whilst individual prevalence could better reflect the risk of zoonotic transmission.

## Introduction

Bovine brucellosis is an important zoonotic bacterial disease caused by *Brucella abortus* (Bamaiyi 2016). According to Du Preez and Du Preez (2018):

[*H*]uman brucellosis is also known as *brucella* fever, abortus fever and undulant fever (when caused by *B. abortus*). The disease in cattle is also known as contagious abortion or simply CA. (n.p.)

It has a wide host range, but infections in domestic cattle have a serious impact on food security, food safety and rural economics in Africa as well as human health (Franc et al. 2018; Godfroid et al. 2011; Makita et al. 2011). The Food and Agriculture Organization, the World Health Organization, and the World Organisation for Animal Health consider brucellosis to be the second most important zoonotic disease in the world, after rabies (Abubakar, Mansoor & Arshed 2012). McDermott, Grace and Zinsstag mentioned in 2013 that there were more than 500 000 cases of human brucellosis every year. Infections are characterised by fever, malaise, urogenital symptoms, anorexia, insomnia, weight loss, headache and joint pains. It is easily confused with malaria and influenza (Bamaiyi 2016; Doganay & Aygen 2003). Brucellosis in humans is under-reported because of vague clinical symptoms, difficulties with laboratory diagnosis and the medical profession’s lack of knowledge about the disease (Bwala et al. 2015; Dean et al. 2012; Franco et al. 2007; Frean et al. 2018; Ibironke et al. 2008; Mukhtar & Kokab 2008). The World Health Organization considers it a neglected zoonosis, because it is seldom prioritised by national and international health systems (World Health Organization 2006). In Africa, bovine brucellosis can have serious economic impacts, resulting from loss of work or income due to illness and disability (Bwala et al. 2015). This impact on human health can be quantified using the disability-adjusted life year, or DALY (Marcotty et al. 2009).

Occupations at risk include farmers, farm-workers, butchers, abattoir workers, dairy personnel, veterinarians, laboratory technicians and animal health officers (Bamaiyi 2016; Bwala et al. 2015; Marcotty et al. 2009). Consumers can be infected by unpasteurised dairy products including butter, milk, cheese and yoghurt (Hesterberg et al. 2008). During a study in Turkey, the sero-prevalence rate was 1.8% in the general population and 6% in high-risk occupational groups (Doganay & Aygen 2003). The sero-prevalence in cattle is closely related to the number of human cases (Tumwine et al. 2015). In Mongolia, a sero-prevalence of 17% was found in rural populations (Tsend et al. 2014).

Routine active sero-surveillance is an essential component in a bovine brucellosis control strategy (Adone & Pasquali 2013; World Organisation for Animal Health 2019). A cattle herd is presumed positive even if only one positive cow is found (D’Orazi et al. 2007). Different authors may compare individual animal and herd prevalence, often in the same publication (Mai et al. 2012). Populations are not being defined – for instance, Ndazigaruye et al. (2018) mentioned that the prevalence of bovine brucellosis in East Africa ranged from 6.5% to 48.0%; however, these estimates were based on several different testing methods, as well as a combination of herd and individual prevalence.

Bovine brucellosis is a controlled animal disease in terms of the South African Animal Diseases Act, 35 of 1984 (repealed by the *Animal Health Act* 7 of 2002) and related regulations (Cloete et al. 2019; South African National Department of Agriculture and Rural Development 2016). The current Brucellosis Scheme in South Africa was formulated in accordance with Regulation 2583 of 9 December 1988, under the *Animal Disease Act*, 35 of 1984. In South Africa, an average of 374 outbreaks were reported annually to the World Organisation for Animal Health between 1996 and 2004 (Hesterberg et al. 2008). Over the same period, the individual prevalence was estimated at 0.75% – 2% in communal cattle in North West Province. In communal areas, where milk and meat is informally harvested, brucellosis is a potential risk to human health (Mokantla et al. 2004).

Although eradication is emphasised to prevent re-introduction in developed countries, information on the prevalence of bovine brucellosis is scarce in developing countries (Godfroid et al. 2004, 2011). In South Africa, provinces manage brucellosis in line with national policies and legislature. According to the Bovine Brucellosis Manual (2016) published by the South African National Department of Agriculture and Rural Development:

[*B*]ovine Brucellosis is a controlled disease in accordance with the Animal Diseases Act (Act 35 of 1984) and the Animal Disease Regulations published in Govt. notice R2026 of 26 September 1986, as well the Bovine Brucellosis Scheme Regulations published in Govt. notice R2483 of 9 December 1988. (p. 49)

Very little has been published on the prevalence of brucellosis in the North West Province since 2004, despite routine sampling of herds by the State Veterinary Services.

The objectives of this study were therefore to analyse available retrospective data from routine serological testing of cattle in the Bojanala Region between 2009 and 2013; and to estimate prevalence trends in herds and individual cattle as well as geographical distribution of outbreaks. In addition, it aimed to obtain data on free vaccination against bovine brucellosis by the State Veterinary Services in the study area over the study period. This information is critical to develop recommendations for future bovine brucellosis control strategies. Specifically those designed to improve rural food safety and security, as well as preventing zoonotic transmission of brucellosis; in the North West Province.

## Research methods and design

A descriptive cross-sectional study design was used to estimate herd and individual sero-prevalence of brucellosis in adult cows (> 18 months old), serologically tested for bovine brucellosis in the Bojanala Province of the North West Province, South Africa between 2009 and 2013.

Bojanala Platinum District Municipality is situated in the north-eastern part of the North West Province. It covers an area of approximately 18 333.38 km^2^. The local municipalities included in the study area were Kgetleng River, Madibeng, Moretele, Moses Kotane and Rustenburg. Although the region is mainly rural with low population densities, it includes the towns of Rustenburg and Brits (Madibeng) which are densely populated. A map of the study area (setting) can be accessed online at https://municipalities.co.za/map/139/bojanala-platinum-district-municipality.

The total population was estimated at about 500 000 and comprised adult cows (> 18 months old) reared in communal, commercial and dairy farming systems in the study area. The sampling strategy was based on all secondary data from existing records of cows bled and tested for bovine brucellosis by the State Veterinary Services between 2009 and 2013 (*n* = 59 663). Samples were transported and tested as prescribed by the World Organisation for Animal Health, because South Africa is a member nation. Serum was tested using the Rose Bengal Test and suspect samples were retested using the Complement Fixation Test (World Organisation for Animal Health 2016). Testing was done at the Potchefstroom and Onderstepoort Veterinary Institute (Agricultural Research Centre) laboratories.

Secondary data on test results were obtained from the regional Directorate of Veterinary Services official laboratory reports. The laboratories reported that 570 samples submitted over the study period were haemolysed and could not be tested. Exclusion criteria were used during data cleaning (using Microsoft Office Professional Excel 2016 software) to remove incomplete or inaccurate data. A method similar to that used by Anka et al. (2013), was used for data entry: owner name, herd number, district, farm name and location, farm co-ordinates, test date, number of animals tested, number of animals positive. Repeated entry of a farm in the course of 1 year was eliminated, to ensure that a herd or farm was not over-represented in the same year. For each year, cases within the test population were analysed to calculate prevalence based on spatial, temporal and farming systems. After cleaning, data from 46 762 cows bled during this ecological cross-sectional survey, were analysed using descriptive statistics.

The prevalence of brucellosis in both individual cows and in herds was estimated as the number of positive cases as a proportion (percentage) of the population (individual animals or herds) tested. A herd was considered infected (or positive) if at least one animal tested positive using both the Rose Bengal Test and Complement Fixation Test (D’Orazi et al. 2007). A herd was defined as a group of animals sharing the same grazing area and/or watering point (Berhe, Belihu & Asfaw 2007). Each of the three farming systems identified were defined, for the purpose of this study, as follows:

- Dairy farms: these were commercial dairy farms where bulk milk samples were routinely sent for milk ring testing and cows were regularly bled for testing.
- Commercial beef farms: these were commercial farmers whose cattle were bred for meat production in the formal sector. The farmers or companies owned or leased the farmland used.
- Communal farms: these were areas mainly in the previous ‘homelands’, where individual farmers herds shared the grazing on communal farmland.

### Ethical consideration

Ethical approval for this study was obtained from the Ethics Committee of the Faculty of Health Sciences, University of Pretoria: Ethics approval number 257/2015.

## Results

Table 1 below shows the number of cows and herds tested per annum in each of the districts in the study area.

**TABLE 1:** Effect of year and district of origin, on number of cows and herds tested annually.

District	Year						
	2009	2010	2011	2012	2013	Mean/district	Std dev
**Individual cows**							
Moretele	1081	6	948	554	802	678.20	318.56
Madibeng	1359	2031	2019	1409	1851	1733.80	233.20
Rustenburg	771	1800	2090	1852	1018	1506.20	407.80
Kgetleng River	4030	5937	4023	5212	3100	4460.40	742.73
Moses Kotane	886	630	1026	1221	1106	973.80	143.87
Mean cows/year	1625.40	2080.80	2021.20	2049.60	1575.40	-	-
Std Dev.	801.53	1285.40	690.20	906.61	600.07	-	-
**Herds**							
Moretele	15	0	19	9	11	10.80	4.20
Madibeng	18	26	16	19	24	20.60	2.93
Rustenburg	10	14	30	18	10	16.40	5.07
Kgetleng River	41	43	45	42	27	39.60	4.20
Moses Kotane	14	10	14	18	14	14.00	1.33
Mean herds/year	19.6	18.6	24.8	21.2	17.2	-	-
Std Dev.	7.13	10.60	8.47	6.93	5.53	-	-

Std Dev., standard deviation.

It can be seen from Table 1, that there was considerable variation between the years (temporal determinant) and districts (spatial determinant) in the number of herds and cows tested.

Table 2 below shows the frequency and proportion of individual animals and herds tested, as well as the sero-prevalence in each of the three cattle farming systems in the study area.

**TABLE 2:** Individual and herd sero-prevalence of brucellosis per farming system over the study period.

Farming system	Communal	Dairy	Commercial beef	Total
Test population	11 815	6696	28 251	46 762
% total tested	25.3	14.3	60.4	100
Positive cases	613	21	852	1436
Individual prevalence (%)	5.19	0.31	3.02	3.18
Number of herds	178	39	290	507
% herds tested	35.10	7.7	57.2	100
Positive herds	69	7	93	169
Herd prevalence (%)	38.76	17.9	32.1	33.33

The highest proportion of herds and individual cows tested was, surprisingly, in commercial beef cattle farming systems, whilst the lowest was in the dairy sector. However, the highest individual and herd prevalence was in the communal sector. It is evident from Table 2, that there was a significant difference between herd and individual prevalence (*p* < 0.05). There is also a significantly lower (*p* < 0.05) mean prevalence in individual dairy cows, than in individual commercial and communal cattle.

Individual and herd prevalence of brucellosis in the five districts of the Bojanala Region of the North West Province are shown in Table 3.

**TABLE 3:** Herd and individual sero-prevalence of brucellosis in the different districts over the entire study period.

District	Moretele	Madibeng	Rustenburg	Kgetleng River	Moses Kotane
Cows tested	3391	8669	7531	22 302	4869
Cows positive	138	168	310	511	359
Individual prevalence (%)	4.07	1.94	4.11	2.29	7.37
Herds tested	54	103	82	198	70
Herds positive	16	25	23	68	37
Herd prevalence (%)	29.63	24.27	28.05	34.34	52.85

From Table 3, it is interesting to note the much higher (*p* < 0.05) prevalence of brucellosis in both herds and individual cows in the Moses Ketana district. In contrast is the far lower prevalence in the Madibeng district. The range of the values for individual prevalence (1.94% – 7.37%) is wider than the range of herd prevalence (24.27% – 52.85%).

Table 4 shows the influence of the year of sampling in each district, for both herd and individual prevalence.

**TABLE 4:** The influence of the year of sampling on the herd and individual sero-prevalence in each district.

Variable	Year				
	2009	2010	2011	2012	2013
**District**					
***Herd prevalence (%)***					
Moretele	40.0	0.0	15.8	33.3	36.4
Madibeng	22.2	19.2	12.5	21.2	41.7
Rustenburg	10.0	14.3	33.3	11.1	80.0
Kgetleng River	34.1	32.6	40.0	38.1	22.2
Moses Kotane	42.9	50.0	57.1	66.7	46.2
***Individual prevalence (%)***					
Moretele	7.22	50.0	0.84	4.15	3.24
Madibeng	1.47	1.92	0.45	2.98	3.13
Rustenburg	3.63	3.44	7.13	0.7	5.7
Kgetleng River	4.12	2.11	2.19	2.17	0.61
Moses Kotane	9.14	8.89	5.17	9.34	4.97

There was a wide variation in both herd and individual prevalence between years. In general, however, the Moses Kotane district retained the highest herd and individual sero-prevalence over all 5 years. In 2010, no herds were tested in Moretele, whilst three of the six individual animals tested in that year were positive, resulting in a distorted estimation of prevalence over the 5-year period.

Other than the test and slaughter policies suggested for control of bovine brucellosis in countries where it is endemic, vaccination is regarded as important (World Health Organization 2019). The North West Province State Veterinary Service provides free vaccination of heifers with Brucella strain 19 (S19) vaccine and also provides free RB51 vaccination of adult cows (Onderstepoort Biological Products, South Africa). Table 5 shows the level of vaccination over the study period in the study area.

**TABLE 5:** Frequency of S19 and RB51 vaccinations given to cattle in the Bojanala Region, 2009–2013.

Year	Eligible animals for free vaccination programme (frequency)	Vaccinated (frequency)	Vaccination rate (%)
2009	68 500	7897	11.53
2010	68 500	4820	7.04
2011	68 500	3517	5.13
2012	68 500	4626	6.75
2013	68 500	3226	4.71

**Total**	**342 500**	**24 086**	**7.03**

It can be seen from Table 5 that only 7% of the eligible cattle in the study area were vaccinated over the study period, although 11.3% were vaccinated in 2009. In 2013, only 4.71% of eligible cattle were vaccinated.

## Discussion

Key findings after the analysis of retrospective data on the sero-prevalence of bovine brucellosis in the Bojanala Region in the North West Province, showed a significant difference between herds and individual cows and that dairy farming systems had a significantly lower spatial and temporal prevalence. The population mean individual prevalence over the whole study period for all three farming systems was 3.18%, with a range of 0.31% – 5.19%. For herd sero-prevalence, the population mean was 33.33%, with a range of 17.9% – 38.76% over the same period. It is therefore very important that publications clearly state whether they are using herd or individual sero-prevalence of brucellosis. For instance, Ndazigaruye et al. (2018) estimated the prevalence of bovine brucellosis in East Africa as being between 6.5% and 48.0%, but acknowledged that this was based on several testing methods, including milk ring tests, culture and serology. It was not mentioned whether these were herd or individual prevalence percentages, which made the findings difficult to interpret or compare.

As there is no human vaccine, the prevention and control of human brucellosis depends on its control in animal hosts (Godfroid et al. 2011). The individual occupational risk for zoonotic diseases such as bovine brucellosis can be estimated using the frequency of exposure multiplied by the dose of the agent. The estimation of exposure requires information about the individual prevalence in cows on a farm or at slaughter. In contrast, cluster prevalence is a better way of reflecting spatial determinants, spread of disease and the geographical distribution of infected herds or flocks, which is important for developing disease control strategies (D’Orazi et al. 2007).

Over the study period, no temporal trends in either individual or herd sero-prevalence were found. More work is needed to discover why the number of cattle tested varied so much from year to year and district to district. However, both individual and herd sero-prevalence in dairy herds were significantly lower in all districts over all years, to that of commercial and communal beef herds. This may be because of the fact that commercial dairy farms are registered and the herds are regularly monitored using both milk ring tests and sero-surveillance.

Another interesting finding was that the number of cattle tested per year was very low in comparison to the estimated population of 500 000. This is partly because of the fact that the test population was limited to breeding cows that were 18 months and older, so calves, steers, heifers and bulls were excluded. According to Du Preez and Malan (2018), approximately 3% – 9% of the heifers that are born from infectious cows may be latently (inconspicuously) infected, although they test serologically negative (no antibodies) until at least 18 months of age. They only present antibodies against *Brucella abortus* that are traceable when they are tested at 4.5 months, or later during their first gestation.

It would have been interesting to discover what proportion of adult breeding cows had been vaccinated. Although the 68 500 cows eligible for subsidised vaccination could give some indication, this number may not include commercial beef and dairy farms. Also, it is not clear what proportion of these were heifers eligible for S19 vaccination.

Godfroid et al (2011) maintain that vaccination is the cornerstone of control programs in livestock. They specifically mention that the S19 and RB51 vaccines are used to prevent bovine brucellosis in cattle. It was clearly shown that even the proportion of eligible cows vaccinated was very low and the trend decreased over time. A confounder was that while Brucella S19 vaccines are only given to heifers between 4 and 8 months of age and are compulsory, RB51 is injected annually in adult cows, so the 68 500 eligible cows may include heifers < 9 months old and these would not be bled for sero-surveillance until they are adult. Also, the data only included free vaccination by the state and not data on cattle vaccinated at the cost of the farmer or cattle owners. Currently, in South Africa, vaccination of heifers between 4 months and 8 months old is compulsory, but vaccination of adult cows with RB51 may only be done with the permission of the Directorate of Veterinary Services, according to the 2016 Interim Brucellosis Manual (Cloete et al. 2019). It would be interesting to find out how many of each type of vaccine is being used and the vaccination status of infected versus non-infected herds. The implications of this study are that more research on the role of vaccination in preventing and controlling the disease is recommended.

**Strengths and limitations:** The main strengths of this investigation were that analysis of the retrospective sero-surveillance data in this study should contribute a good baseline for designing future bovine brucellosis control strategies in the North West Province. The far lower prevalence of bovine brucellosis in dairy cattle points the way to developing better methods of disease control in communal and commercial beef cattle. This was a descriptive cross-sectional study investigating the spatial and temporal aspects of brucellosis in different farming systems, and the limitations of analysing non-parametric data emanating from the field surveillance of notifiable livestock diseases are well known (Food and Agriculture Organization 2011; Thrusfield 2018).

The implications of this study are that more research on the role of vaccination in preventing and controlling the disease is recommended. The economic aspects of state subsidised sero-surveillance and vaccination were also not explored, yet this could have influenced the relatively low proportion of cows bled and vaccinated between 2009 and 2013 in the study area.

## Conclusion

The study analysed available retrospective data from routine serological testing of cattle in the Bojanala Region, North West Province between 2009 and 2013 and estimated prevalence trends in herds and individual cattle, as well as geographical distribution of outbreaks. Data on state-subsidised vaccination against bovine brucellosis in the study area over the study period were also presented. It was concluded that the individual prevalence of bovine brucellosis was significantly lower than herd prevalence over all 5 years, in all districts of the Bojanala Region. It was also found that there was a very significant difference between herd and individual prevalence in all three cattle farming systems investigated. It is therefore strongly recommended that future publications should always state whether the prevalence quoted is for individual cows or for herds.
